# Electrophysiological and Morphological Features of Rebound Depolarization Characterized Interneurons in Rat Superficial Spinal Dorsal Horn

**DOI:** 10.3389/fncel.2021.736879

**Published:** 2021-09-21

**Authors:** Mengye Zhu, Yi Yan, Xuezhong Cao, Fei Zeng, Gang Xu, Wei Shen, Fan Li, Lingyun Luo, Zhijian Wang, Yong Zhang, Xuexue Zhang, Daying Zhang, Tao Liu

**Affiliations:** ^1^Department of Pain Medicine, The First Affiliated Hospital of Nanchang University, Nanchang, China; ^2^Institute of Pain Medicine, Jiangxi Academy of Clinical and Medical Sciences, Nanchang, China; ^3^Center for Experimental Medicine, The First Affiliated Hospital of Nanchang University, Nanchang, China

**Keywords:** rebound depolarization, spinal dorsal horn, substantia gelatinosa neuron, morphology, electrophysiology, primary afferent input

## Abstract

Substantia gelatinosa (SG) neurons, which are located in the spinal dorsal horn (lamina II), have been identified as the “central gate” for the transmission and modulation of nociceptive information. Rebound depolarization (RD), a biophysical property mediated by membrane hyperpolarization that is frequently recorded in the central nervous system, contributes to shaping neuronal intrinsic excitability and, in turn, contributes to neuronal output and network function. However, the electrophysiological and morphological properties of SG neurons exhibiting RD remain unclarified. In this study, whole-cell patch-clamp recordings were performed on SG neurons from parasagittal spinal cord slices. RD was detected in 44.44% (84 out of 189) of the SG neurons recorded. We found that RD-expressing neurons had more depolarized resting membrane potentials, more hyperpolarized action potential (AP) thresholds, higher AP amplitudes, shorter AP durations, and higher spike frequencies in response to depolarizing current injection than neurons without RD. Based on their firing patterns and morphological characteristics, we propose that most of the SG neurons with RD mainly displayed tonic firing (69.05%) and corresponded to islet cell morphology (58.82%). Meanwhile, subthreshold currents, including the hyperpolarization-activated cation current (I_*h*_) and T-type calcium current (I_*T*_), were identified in SG neurons with RD. Blockage of I_*h*_ delayed the onset of the first spike in RD, while abolishment of I_*T*_ significantly blunted the amplitude of RD. Regarding synaptic inputs, SG neurons with RD showed lower frequencies in both spontaneous and miniature excitatory synaptic currents. Furthermore, RD-expressing neurons received either Aδ- or C-afferent-mediated monosynaptic and polysynaptic inputs. However, RD-lacking neurons received afferents from monosynaptic and polysynaptic Aδ fibers and predominantly polysynaptic C-fibers. These findings demonstrate that SG neurons with RD have a specific cell-type distribution, and may differentially process somatosensory information compared to those without RD.

## Introduction

Rebound depolarization (RD) is a transient membrane depolarization (sometimes accompanied by a series of spikes) following hyperpolarizing pulses. It has been observed in various brain regions, which include the hippocampus (Surges et al., 2006), auditory midbrain (Sun et al., 2020), medial prefrontal cortex (Kurowski et al., 2018), thalamus (Wang et al., 2016; Zhu et al., 2018), and deep spinal dorsal horn (Rivera-Arconada and Lopez-Garcia, 2015), among others. RD substantially relies on channels with hyperpolarization-dependent activation or de-inactivation features. Hyperpolarization-activated cation current (I_*h*_), a mixed inward current consisting of sodium and potassium ions, mediated by hyperpolarization-activated cyclic nucleotide-gated (HCN) channels has been confirmed to be able to regulate RD. In addition, T-type calcium channel-induced current (I_*T*_) is another vital ionic conductance supporting RD generation (Sangrey and Jaeger, 2010; Engbers et al., 2011; Duan et al., 2018). Functionally, RD has been proposed as an inhibition-excitation converter transforming inhibitory inputs into excitatory signals (Sanchez-Vives and McCormick, 2000; Tadayonnejad et al., 2009; Pedroarena, 2010).

Lamina II of the spinal dorsal horn, namely substantia gelatinosa (SG), is an indisputably important component of the pain pathway. The SG is referred to as the “central gate” since it converses inputs from the primary afferents, local circuit interneurons, and endogenous descending tracts, and may therefore be essential in transmitting and modulating nociceptive information from the periphery (Todd, 2010; Duan et al., 2018). Alterations in SG neuronal excitability have been implicated as catalysts for the development and maintenance of pathological pain (Balasubramanyan et al., 2006; Kuner, 2010; Feng et al., 2019). The SG is composed of excitatory and inhibitory interneurons with substantial electrophysiological and morphological heterogeneity (Grudt and Perl, 2002; Maxwell et al., 2007; Yasaka et al., 2010; Graham and Hughes, 2020). RD is a striking biophysical property of SG neurons (Tadros et al., 2012; Hu et al., 2016). Nevertheless, whether SG neurons with RD display distinct morphological features, intrinsic electrophysiological properties, and input of afferent fibers has not yet been well studied. In addition, the ionic basis for RD in SG neurons also remains to be elucidated.

Here, we used the whole-cell patch-clamp technique to record passive and active membrane properties from SG neurons, which were further categorized to the neurons with RD and without RD. Interestingly, we found that a majority of the SG neurons with RD showed tonic firing, and a *post hoc* morphological study confirmed that most of the RD-expressing neurons exhibited islet morphology. Besides, RD neurons also presented significant differences in intrinsic neural excitability, as well as spontaneous, miniature, and evoked excitatory synaptic transmission. Additionally, we confirmed that I_*h*_ and I_*T*_ are vital ionic contributions to RD responses in SG neurons. Therefore, our results may provide new insights for unraveling the role of RD in pain processing.

## Materials and Methods

### Animals

Sprague-Dawley (SD) rats (4–6 weeks old) of both sexes obtained from the Animal Center of Nanchang University were used. Rats were housed in same-sex groupings (4–5 animals per cage) in an air-conditioned room maintained at 24 ± 1°C and 50–60% humidity, under a 12:12 light/dark cycle (lights on at 7 a.m.). They had *ad libitum* access to food and water. All animal procedures were performed according to methods approved by the Institutional Animal Care and Use Committee of Nanchang University. Maximal efforts were made to minimize animal pain or discomfort and the number of animals used.

### Spinal Cord Slice Preparation

Acute spinal cord slices were prepared as previously described (Wu et al., 2018; Zhu et al., 2019). Briefly, rats were deeply anesthetized with urethane (1.5 g/kg, i.p.), and were then transcardially perfused with an ice-cold sucrose-based artificial cerebrospinal fluid (sucrose-ACSF) containing (in mM): 204 sucrose, 2.5 KCl, 3.5 MgCl_2_, 0.5 CaCl_2_, 1.25 NaH_2_PO_4_, 0.4 ascorbic acid, 2 sodium pyruvate, 11 D-glucose, 25 NaHCO_3_, and 1 kynurenic acid. The lumbosacral section of the vertebral column was quickly removed and placed into the same solution. After laminectomy and removal of the dura mater, the spinal cord was mounted on a vibratome (VT1000S, Leica, Nussloch, Germany) cutting stage. Sucrose-ACSF was preoxygenated with 95% O_2_ and 5% CO_2_ for at least 30 min before use. Parasagittal slices (400–600 μm) with or without dorsal root (DR) (8–12 mm long) attached were prepared and kept in an incubator at 32°C for at least 30 min before electrophysiological recording in carbonated standard ACSF. The standard ACSF consisted of (in mM) 117 NaCl, 3.6 KCl, 2.5 CaCl_2_, 1.2 MgCl_2_, 1.2 NaH_2_PO_4_, 25 NaHCO_3_, 11 D-glucose, and 2 sodium pyruvate.

### *In vitro* Electrophysiological Recordings

Spinal cord slices were transferred into a recording chamber and perfused continuously with carbogenated standard ACSF at a flow rate of 2–4 ml/min. Experiments were performed at room temperature (RT) under visual guidance using an Olympus microscope (BX51WI, Olympus Corp., Tokyo, Japan) and an IR-DIC camera (IR-1000, Dage-MTI, Michigan City, IN, United States). Patch-clamp recordings in the whole-cell configuration were made using an EPC-10 amplifier and Patchmaster software (HEKA Electronics, Lambrecht, Germany). Patch pipettes (3–6 MΩ) were pulled from borosilicate glass capillaries (1.5 mm OD, 1.12 mm ID, World Precision Instruments, Sarasota, FL, United States) with a Sutter P-97 puller (Sutter Instruments, Novato, CA, United States). The internal pipette solution contained (in mM) 130 K-gluconate, 5 KCl, 10 Na_2_-phosphocreatine, 0.5 EGTA, 10 HEPES, 4 Mg-ATP, and 0.3 Li-GTP (pH 7.3 adjusted with KOH, 295 mOsm).

Only neurons with a resting membrane potential (RMP) more negative than −45 mV and showing overshooting action potentials (APs) (i.e., exceeding 0 mV) were included for this study. The series resistance was typically between 10 and 30 MΩ, and cells in which the series resistance changed by more than 20% were discarded from further analysis. No correction for liquid junction potential (approximate 15 mV) was made in this study. All data analyses were performed using Clampfit (Molecular Devices, CA, United States) and Mini Analysis software (Synaptosoft Inc., GA, United States).

Membrane capacitance (Cm) was estimated from the area under the transient capacitive current evoked with a 5-mV depolarizing pulse by Patchmaster software in real-time automatically. Neuronal RMP was recorded within 20 s after the break-in. A hyperpolarizing voltage step was used to estimate the input resistance (Rin) of the tested SG neuron, while negative current pulses (−120 and −140 pA, 1 s) were applied to generate RD responses. The firing pattern of each neuron was determined with a series of depolarizing current pulses (20–140 pA in 20 pA increments) of 1-s duration in the current-clamp mode. The spike adaptation index was obtained by dividing the average of the first two interspike intervals (ISIs) by that of the last two ISIs (Ha et al., 2016). A subthreshold current was evoked by a series of hyperpolarizing voltage steps from −50 to −130 mV in 10 mV decrements (duration 1 s) at a −50 mV holding potential in the voltage-clamp mode.

To investigate the primary afferent inputs, neurons were voltage-clamped at −70 mV. DR-evoked excitatory postsynaptic currents (eEPSCs) were initiated by a constant current source of a pulse (duration 0.1 ms) at a frequency of 0.05 Hz delivered through a suction electrode. The stimulation intensities were set at 50 and 100 μA for the activation of Aδ fibers, 500 μA and 1 mA for the activation of C fibers using a stimulator (Master 8, AMP Instruments Ltd., Israel) and a stimulus isolator (ISO-Flex, AMP Instruments Ltd., Israel). Neurons without an evident monosynaptic response at 1 mA were subsequently stimulated at 3 and/or 5 mA. The Aδ or C-afferent-mediated responses evoked by DR stimulation were distinguished based on the stimulus intensity and the conduction velocity of afferent fibers (<0.8 m/s for C fibers and >1.0 m/s for Aδ fibers). Evoked-EPSCs were judged to be monosynaptic if there were no failures during subsequent repeated stimulation (20 times at 2 Hz for Aδ and 1 Hz for C fibers) and if their latency remained constant in repetitive trials as reported previously (Cui et al., 2016; Iwagaki et al., 2016).

### *Post hoc* Morphological Identification

For morphological identification, an internal pipette solution containing 0.05% neurobiotin 488 was used. After maintaining the stable whole-cell patch-clamp configuration for at least 20 min, the electrode was gently withdrawn from the targeted neuron, and the slice was then fixed at RT for 1 h and then at 4°C overnight in 4% paraformaldehyde in 0.1 M PB (pH 7.4). Following fixation, slices were rinsed in PBS three times and treated with 50% ethanol for 30 min. After rinsing in PBS, the slices were mounted onto slides with a non-fluorescing mounting medium. Neurobiotin-filled neurons were identified and reconstructed under 20× magnification, 1.0–1.5 zoom, and 1.5 μm stack using a confocal microscope (LSM 700, Zeiss, Germany). Neurons were morphologically grouped on the basis of the following parameters regarding their dendritic arborizations (RC: rostro-caudal extent of dentrites, DV: dorsal-ventral expansion of dendrites, SR, SC, SD, SV: dendrites spread from center of the soma to rostral, caudal, dorsal and ventral limit, respectively) (Grudt and Perl, 2002; Yasaka et al., 2007). As long as the value of RC/DV exceeded 3.5, cells could be classified as an islet or a central cell. Within this group, islet cells exhibited abundant dendrites elongated in the RC dimension (>400 μm), whilst central neurons possessed strikingly shorter RC extents (<400 μm) of their dendric arbors. Neurons with RC/DV less than 3.5 were identified as vertical or radial cells. Vertical neurons generally had a predominantly ventrally oriented dendritic geometry with SV/SD > 3.5. If the ratio of SV/SD was less than 3.5, the neurons were defined as a radial neuron. Neurons differed from the four morphological categories were identified as unclassified cell.

### Chemical Compounds

ZD7288 and tetrodotoxin (TTX) were purchased from Tocris Bioscience, 3,5-dichloro-*N*-[1-(2,2-dimethyl-tetrahydro-pyran-4-ylmethyl)-4-fluoro-piperidin-4-ylmethyl]-benzamide (TTA-P2) and neurobiotin 488 were obtained from Alomone Labs and Vector, respectively. All other reagents were obtained from Sigma-Aldrich. All chemicals were dissolved in distilled water except for TTA-P2, which was prepared in 0.1% DMSO. Chemical compounds were applied at final concentrations by dissolving them in standard ACSF to achieve the necessary final concentrations of antagonism established in previous studies (Peng et al., 2017; Wu et al., 2018).

### Statistical Analysis

Statistical data analysis was conducted with GraphPad Prism 7 (GraphPad Software, La Jolla, CA, United States). For data displaying normal distribution and homogeneity of variance indicated by the Shapiro–Wilk normality test and Levene test respectively, ANOVA or *t*-test was used as appropriate. Numerical data are presented as mean ± SEM. Otherwise, the Mann Whitney Wilcoxon test or Wilcoxon matched-pairs signed-rank test was used for non-parametric statistical comparisons. Data of AP number were analyzed by two-way ANOVA followed by the Bonferroni *post hoc* test. The Chi-squared test or Fisher’s exact test was applied to analyze the difference between groups regarding the proportions of the firing patterns, subthreshold currents, and morphological categories. Differences were considered significant when *p* < 0.05.

## Results

**FIGURE 1:**
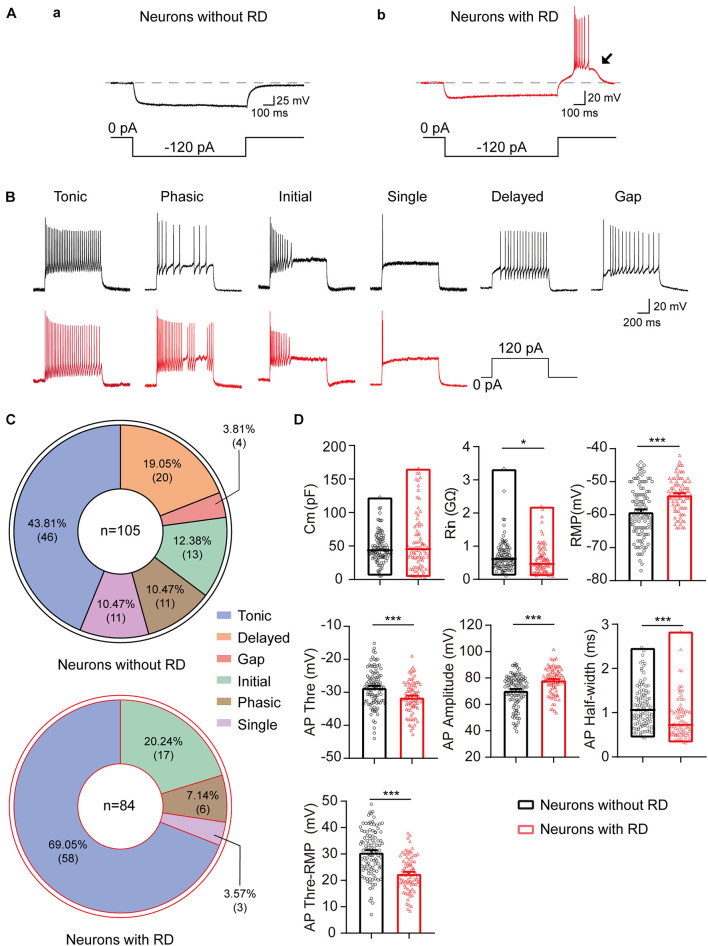
Electrophysiological properties of SG neurons with or without RD from rats. **(A)** SG neurons without **(a)** or with RD **(b)** were classified by a hyperpolarizing current pulse (−120 pA, 1 s). The black arrow indicates the presence of RD. **(B)** Representative traces showing typical firing patterns recorded from SG neurons without (black) or with RD (red). **(C)** Pie graphs showing the proportion of the different firing patterns in SG neurons lacking RD and those expressing RD. In total, 105 and 84 cells were recorded in each group, respectively. **(D)** Summary of the passive and active membrane properties of RD-expressing SG neurons (red) and those lacking RD (black). RD, rebound depolarization; Cm, membrane capacitance; Rin, input resistance; RMP, resting membrane potential; AP, action potential. *p < 0.05, ***p < 0.001.

A total of 189 SG neurons were recorded from parasagittal spinal cord slices of SD rats. As shown in Figure 1A, RD exhibits a pronounced depolarization that often results in spikes at the end of a negative current pulse. According to their response to this current, SG neurons are divided into two groups: with RD (Figure 1Aa) and without RD (Figure 1Ab). The proportions were 44.44% (84/189) and 55.56% (105/189), respectively.

### Intrinsic Passive and Active Membrane Properties

As shown previously, the discharge patterns of SG neurons were highly heterogeneous, including tonic firing, phasic firing, initial firing, single firing, delayed firing, and gap firing (Ruscheweyh and Sandkuhler, 2002; Zhu et al., 2019). We found that the distribution of the discharge patterns differed between the two groups (*p* < 0.001). All six firing categories were encountered in SG neurons lacking RD: tonic (43.81%), phasic (10.47%), initial (12.38%), single (10.47%), delayed (19.05%), and gap (3.81%) (Figures 1B,C). However, delayed firing and gap firing were not identified in SG neurons with RD, of which 69.05% displayed a tonic firing. The proportions of phasic, initial, and single firing in SG neurons with RD were 7.14, 20.24, and 3.57%, respectively (Figures 1B,C).

The results of the passive and active membrane properties of SG neurons with or without RD are summarized in Figure 1D and Table 1. There was no significant difference in Cm. However, the RMP, Rin, and properties of AP of the two populations were significantly different. Neurons with RD had a more depolarized RMP versus those without RD (−54.05 ± 0.58 mV vs −59.18 ± 0.77 mV, *p* < 0.001). In addition, RD-expressing neurons showed a smaller Rin (0.59 ± 0.05 GΩ vs 0.71 ± 0.05 GΩ, *p* = 0.013), a more hyperpolarized AP threshold (−31.53 ± 0.53 mV vs −28.59 ± 0.54 mV, *p* < 0.001), a shorter AP half-width (0.87 ± 0.05 ms vs 1.14 ± 0.05 ms, *p* < 0.001), a smaller potential difference between AP threshold and RMP (22.52 ± 0.71 mV vs 30.59 ± 0.84 mV, *p* < 0.001), and a larger AP amplitude (78.13 ± 1.09 mV vs 70.52 ± 1.17 mV, *p* < 0.001) compared to SG neurons without RD. These results suggest that RD-expressing SG neurons exhibit higher neuronal membrane excitability.

**TABLE 1:** Comparison of intrinsic membrane properties of SG neurons with or without RD.

Parameter	Neurons without RD	Neurons with RD	*p* Value
Cm (pF)	47.24 ± 2.11	54.80 ± 4.60	0.948
Rin (GΩ)	0.71 ± 0.05	0.59 ± 0.05*	0.013
RMP (mV)	−59.18 ± 0.77	−54.05 ± 0.58***	<0.001
APThreshold (mV)	−28.59 ± 0.54	−31.53 ± 0.53***	<0.001
APAmplitude (mV)	70.52 ± 1.17	78.13 ± 1.09***	<0.001
APHalf-width (ms)	1.14 ± 0.05	0.87 ± 0.05***	<0.001
APThreshold - RMP (mV)	30.59 ± 0.84	22.52 ± 0.71***	<0.001

*Values given as mean ± SEM. RD, rebound depolarization; Cm, membrane capacitance; Rin, input resistance; RMP, resting membrane potential; AP, action potential.*

*^∗^p < 0.05.*

*^∗∗∗^p < 0.001.*

**FIGURE 2:**
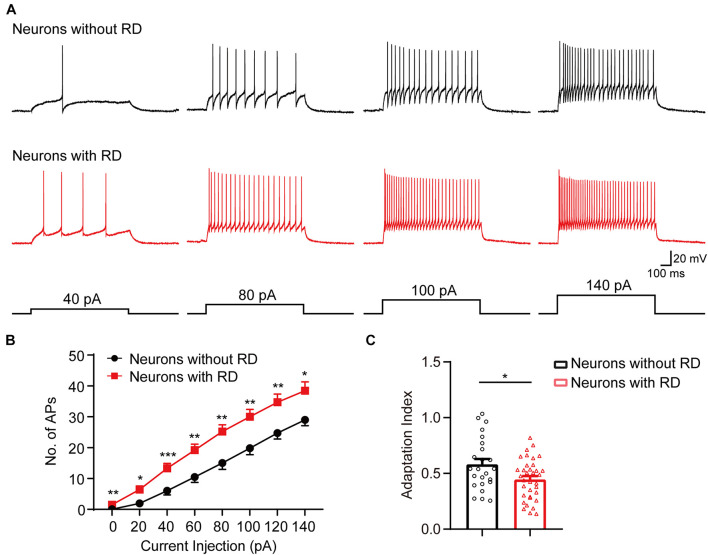
SG neurons with RD that have a tonic-firing pattern exhibit elevated spike frequencies. **(A)** Representative current-clamp traces showing the responses of SG neurons without (black) or with (red) RD to the indicated depolarizing currents of 40, 80, 100, and 140 pA. **(B)** Population data showing the number of spikes of SG neurons expressing or lacking RD with a tonic-firing pattern. **(C)** Summary bar graphs showing that neurons with RD exhibited an elevated spike-frequency adaptation. RD, rebound depolarization; AP, action potential. *p < 0.05, **p < 0.01, ***p < 0.001.

As tonic-firing neuron has repeated APs at relatively regular rates and is the highest proportion in both types of neurons, we next compared the responses of tonic-firing neurons to depolarized current injections (Figure 2A). Tonic-firing neurons from the group with RD showed significantly higher spike frequencies compared to those lacking RD (Figure 2B), suggesting that the former was more excitable. Furthermore, we found that the spike adaptation index was smaller in neurons with RD than those without RD (0.45 ± 0.03 vs 0.58 ± 0.05, *p* = 0.015) (Figure 2C).

### Subthreshold Currents

**FIGURE 3:**
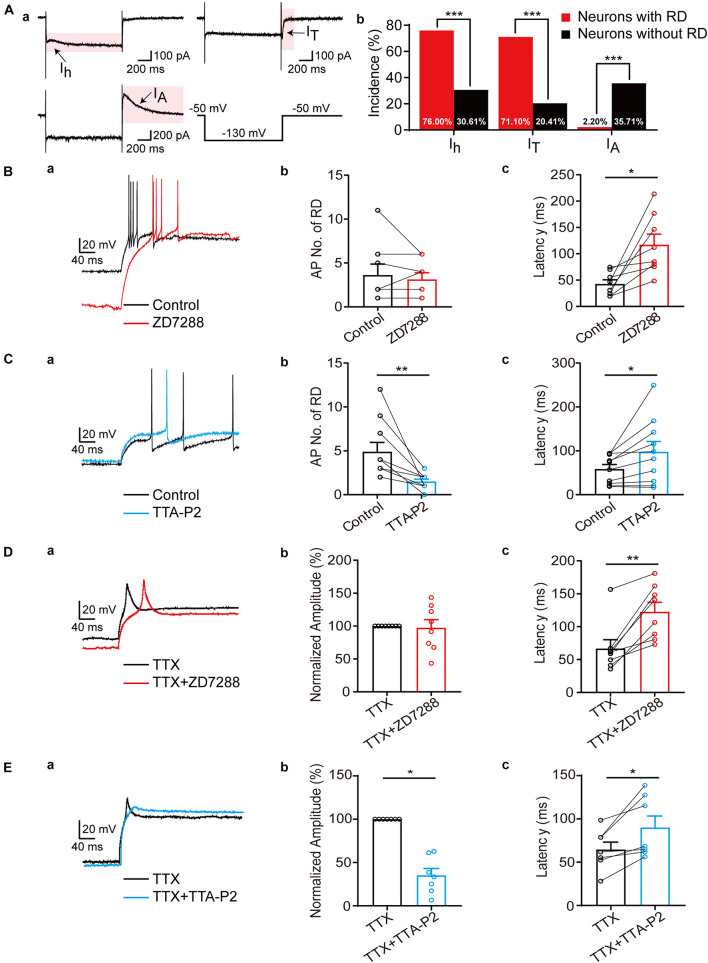
Differential modulation of I_*h*_ and I_*T*_ to RD in SG neurons. **(A)** The subthreshold currents recorded from SG neurons. **(a)** Representative traces of the subthreshold currents (I_*h*_, I_*T*_, and I_*A*_) recorded in SG neurons. **(b)** The subthreshold currents were differentially distributed among SG neurons with (red) or without (black). **(B)** RD in SG neurons was modulated by I_*h*_. **(a)** Representative traces of RD elicited by a hyperpolarizing current pulse (−120 pA, 1 s) in control (black) and with 10 μM ZD7288 (red) in the absence of TTX. **(b,c)**. Summary of the blockage effect of ZD7288 on AP No. **(b)** and first spike latency of RD **(c)** in the absence of TTX. **(C)** RD in SG neurons was modulated by I_*T*_. **(a)** Representative traces of RD in control (black) and with 10 μM TTA-P2 (blue) in the absence of TTX. **(b,c)** Summary of the blockage effect of TTA-P2 on AP No. **(b)** and first spike latency of RD **(c)** in the absence of TTX. **(D)** Effect of ZD7288 on RD of SG neurons in the presence of TTX. **(a)** Representative traces of RD response in TTX (black) and in TTX along with 10 μM ZD7288 (red) in the presence of TTX. **(b,c)** Summary of the blockage effect of ZD7288 on AP No. **(b)** and first spike latency of RD **(c)**. **(E)** Effect of TTA-P2 on RD of SG neurons in the presence of TTX. **(a)** Representative traces of RD response in TTX (black) and in TTX along with 10 μM TTA-P2 (blue) in the presence of TTX. **(b,c)** Summary of the blockage effect of TTA-P2 on AP No. **(b)** and first spike latency of RD **(c)**. RD, rebound depolarization; I_*h*_, hyperpolarization-activated cation current; I_*T*_, T-type calcium current; I_*A*_, A-type current; AP, action potential; TTX, tetrodotoxin. *p < 0.05, **p < 0.01, ***p < 0.001.

Based on previous studies, three subthreshold currents, I_*h*_, I_*T*_, and A-type currents (I_*A*_) were identified in SG neurons (Figure 3Aa; Tadros et al., 2012). In this study, we found that the expression and distribution of the subthreshold currents in RD-lacking SG neurons varied markedly from those in neurons with RD (*p* < 0.001) (Figure 3Ab). Overall, 76.00 and 71.11% of SG neurons with RD expressed I_*h*_ and I_*T*_, respectively. I_*A*_ was rarely encountered (2.22%) in this population. However, in neurons without RD, the prevalence rates of I_*h*_ and I_*T*_ dropped to a relatively low level (30.61 and 20.41%, respectively), while I_*A*_ became the dominant subthreshold current accounting for 35.71%. Measurement of the amplitude and time constant of subthreshold currents showed that no significant differences were observed regarding the activation and inactivation time constants of I_*T*_ between RD-positive and RD-negative neurons. However, RD-expressing neurons possessed faster time constant of I_*h*_, larger current amplitude and density of both I_*h*_ and I_*T*_ (Supplementary Figure 3 and Tables 1, 2).

**TABLE 2:** Primary afferent input onto SG neurons with or without RD.

	Neurons without RD (*n* = 24)	Neurons with RD (*n* = 27)
Aδ-fiber monosynaptic	2 (8.33%)	10 (37.04%)
Aδ-fiber polysynaptic	13 (54.17%)	5 (18.52%)
C-fiber monosynaptic	0 (0.00%)	4 (14.81%)
C-fiber polysynaptic	20 (83.33%)	17 (62.96%)
Convergent Aδ- and C-fiber	11 (45.83%)	9 (33.33%)

*RD, rebound depolarization.*

Because I_*h*_ along with I_*T*_ has been proposed to be the major ionic basis responsible for RD in neurons of the deep cerebellar nuclei, periventricular preoptic area, etc. (Engbers et al., 2011; Zhang et al., 2013), we next studied the effects of I_*h*_ and I_*T*_ on RD in SG neurons. Bath application of ZD7288 (10 μM), a specific HCN channel blocker, increased RD latency from 42.64 ± 7.93 ms to 117.17 ± 20.11 ms (*p* = 0.011) but did not affect the number of spikes generated (3.63 ± 1.25 vs 3.13 ± 0.77, *p* = 0.750) (Figure 3B). In addition, the blockage of I_*T*_ by TTA-P2 (10 μM) markedly decreased RD spiking number from 4.90 ± 1.06 to 1.50 ± 0.27 (*p* = 0.002), and increased RD latency from 58.59 ± 10.07 ms to 97.79 ± 23.47 ms (*p* = 0.034) (Figure 3C).

In attempt to better addressing the influence of I_*h*_ or I_*T*_ on RD properties, TTX (0.5 μM) was applied to the bath solution to eliminate rebound discharge. In the presence of TTX, blockage of I_*h*_ led to a substantial delay in latency (66.71 ± 13.42 ms to 122.55 ± 14.38 ms, *p* = 0.008) without alterations of the RD amplitude (Figure 3D). In addition, the blocking effect of TTA-P2 almost eliminated RD. As illustrated in Figure 3E, the RD amplitude dropped by approximately 64.97% ± 8.03% (*p* = 0.016), and the RD latency raised from 64.67 ± 8.55 ms to 90.05 ± 13.32 ms (*p* = 0.020) with bath application of TTA-P2(Figure 3E). Taken together, these results suggest that RD in SG neurons is mainly mediated by I_*T*_, while I_*h*_ facilitate its onset.

### Properties of Spontaneous and Miniature Excitatory Postsynaptic Currents

**FIGURE 4:**
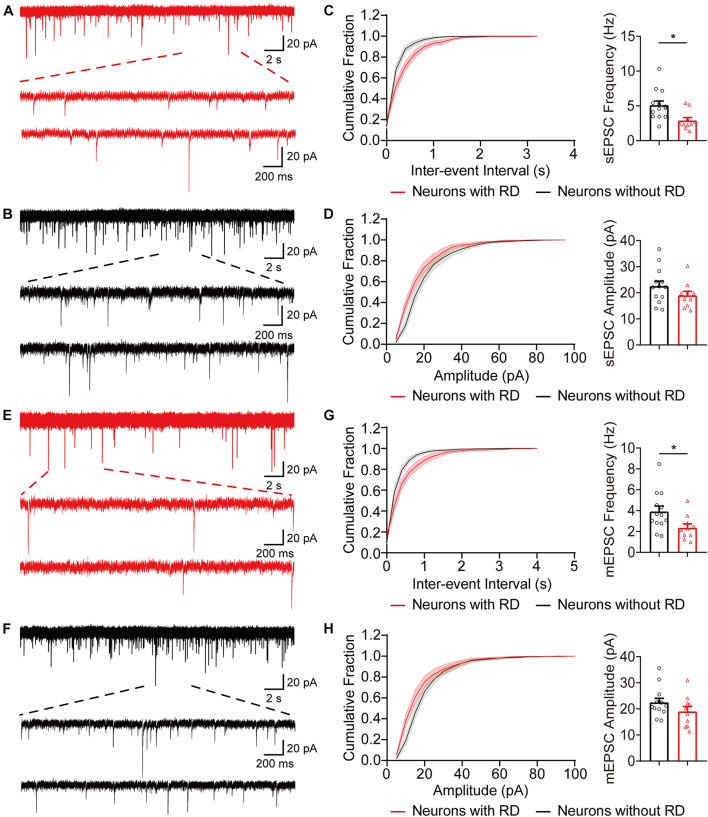
Excitatory synaptic inputs differ in SG neurons with or without RD. **(A,B)** Exemplar traces of sEPSCs in SG neurons expressing **(A)** or lacking **(B)** RD. The bottom panels show the proportional enlargements corresponding to the upper traces. **(C,D)** Cumulative probability and summary bar graphs of the inter-event interval **(C)** as well as the amplitude **(D)** showing a lower frequency of sEPSCs in neurons with RD. **(E,F)** Typical traces of mEPSCs in RD-expressing **(E)** or RD-lacking **(F)** SG neurons. **(G,H)** Summarized data showing that RD-expressing neurons had a lower mEPSCs frequency compared to those lacking RD **(G)**, while mean amplitudes of mEPSCs from the two populations were comparable **(H)**. sEPSC, spontaneous excitatory postsynaptic current; mEPSC, miniature excitatory postsynaptic current. *p < 0.05.

Next, we examined whether SG neurons with RD exhibited any difference in the excitatory synaptic communication compared to those lacking RD. As shown in Figure 4, the frequencies of sEPSCs recorded from neurons with RD tended to be approximately 56% of those without RD (2.89 ± 0.43 Hz vs 5.10 ± 0.60 Hz, *p* = 0.010), while the difference of amplitudes of sEPSCs was not significant (19.02 ± 1.57 pA vs 22.58 ± 1.91 pA, *p* = 0.182) (Figures 4A–D). SG neurons with and without RD did not differ in mEPSCs amplitudes (19.02 ± 1.91 pA vs 22.50 ± 1.56 pA, *p* = 0.169), but neurons with RD had considerably lower mEPSCs frequencies compared with neurons lacking RD (2.36 ± 0.37 Hz vs 3.91 ± 0.52 Hz, *p* = 0.032) (Figures 4E–H).

### Excitatory Inputs From Both Aδ and C Primary Afferents

**FIGURE 5:**
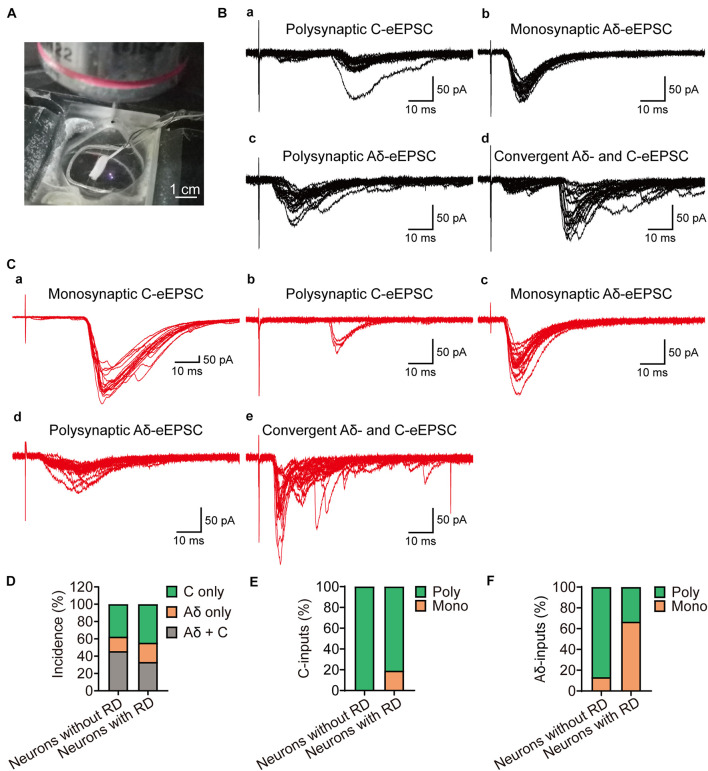
Primary afferent innervation onto SG neurons with or without RD examined by dorsal root stimulation. **(A)** Exemplar image shows a customized suction electrode for the stimulation onto a DR that attached with a parasagittal spinal cord slice. **(B)** Representative traces of Aδ- and C-fiber induced EPSCs of SG neurons without RD, evoked by high-frequency stimulation. **(a)** Polysynaptic C-fiber evoked eEPSC, **(b)** Monosynaptic Aδ-fiber mediated eEPSC, **(c)** Polysynaptic Aδ-fiber mediated eEPSC, **(d)** Convergent Aδ- and C-fiber mediated inputs onto SG neurons without RD. **(C)** Representative traces of Aδ- and C-fiber-evoked EPSCs recorded from SG neurons with RD. **(a)** Monosynaptic C-fiber evoked eEPSC, **(b)** Polysynaptic C-fiber evoked eEPSC, **(c)** Monosynaptic Aδ-fiber mediated eEPSC, **(d)** Polysynaptic Aδ-fiber mediated eEPSC, **(e)** Convergent Aδ- and C-fiber mediated inputs onto SG neurons without RD. **(D)** Quantification of Aδ- and C-fiber-induced eEPSCs onto SG neurons with or without RD by electrical stimuli. **(E)** Quantification of mono- and polysynaptic C-fiber mediated responses in different groups upon DR stimulation. **(F)** Quantification of mono- and polysynaptic Aδ-fiber mediated responses in different groups upon DR stimulation. RD, rebound depolarization; DR, dorsal root; eEPSC, evoked excitatory postsynaptic current.

As SG neurons receive different inputs of Aδ and C primary afferents, we next examined the inputs onto SG neurons with or without RD (Figure 5A). DR stimulation resulted in eEPSCs in 27 (out of 39) RD-expressing and 24 (out of 34) RD-lacking neurons, respectively (Figures 5B,C). RD-expressing neurons received either mono- or polysynaptic inputs from Aδ- and/or C-fibers (Figure 5C). C-fiber-mediated EPSCs were observed in 77.78% (21/27) of the cells. Repetitive stimulation at 1 Hz revealed that in most cases, there was a polysynaptic component (80.95%, 17/21) (Figures 5Ca,b,E). In addition, 45.56% (15/27) of RD-expressing neurons exhibited eEPSCs produced by Aδ fibers, of which 66.67% (10/15) appeared to be monosynaptic (Figures 5Cc,d,F). Conversely, for neurons lacking RD, DR stimulation evoked polysynaptic but not monosynaptic C-fiber-induced EPSCs in 83.33% (20/24) of neurons (Figures 5Ba,D,E). In addition, we observed Aδ-fiber-mediated EPSCs in 62.50% (15/24) of RD-lacking cells, with 13.33% (2/15) and 86.67% (13/15) of these Aδ-responsive cells displaying monosynaptic and polysynaptic responses, respectively (Figures 5Bb,c,F). The conduction velocity, rise time, decay time and mean amplitude of eEPSCs were comparable between neurons with or without RD except for the amplitude of Aδ-fiber-evoked responses (Supplementary Tables 3, 4). RD-expressing neurons possessed larger Aδ-fiber-mediated EPSCs (235.04 ± 43.15 pA vs 86.86 ± 17.95 pA, *p* = 0.003) (Supplementary Table 4).

### Morphological Characterization

**FIGURE 6:**
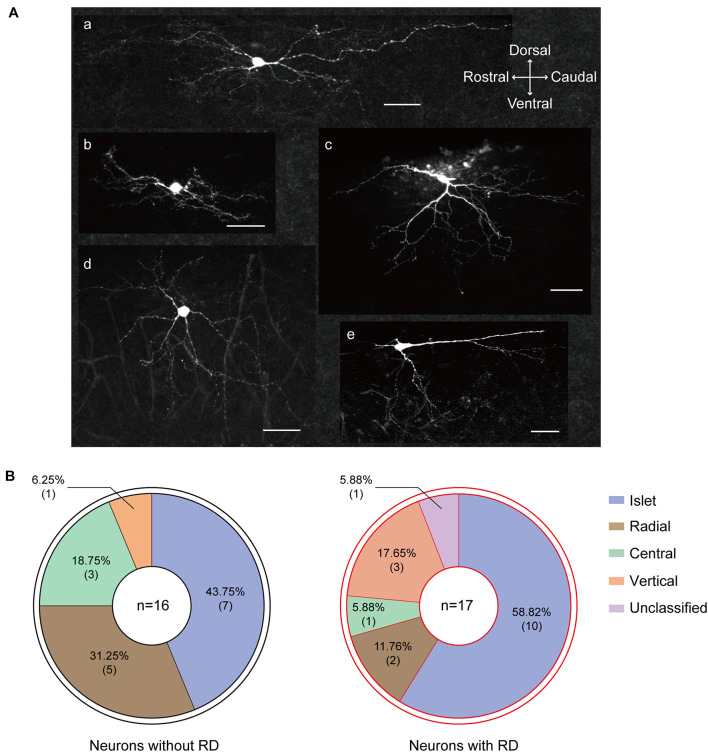
Morphological heterogeneity of RD-expressing SG neurons. **(A)** Confocal images of SG neurons with RD labeled with biocytin. **(a)** islet, **(b)** central, **(c)** vertical, **(d)** radial, **(e)** unclassified. **(B)** Proportion of SG neurons with **(right)** or without **(left)** RD exhibiting different morphologies. RD, rebound depolarization.

It has been well established that SG neurons exhibit complex and diverse morphologies. In this study, 46 recordings were attempted to recover morphology among which 33 were finally morphologically identified, and examples were illustrated in Figure 6A. All five morphological categories (islet, radial, central, vertical, and unclassified) were observed in the examined neurons, and the morphology distributions between the two groups were statistically similar (Figure 6B) (*p* = 0.332). Islet cell was clearly the type with the highest prevalence encountered in both RD-expressing and -lacking SG neurons (58.82 and 43.75%, respectively). Given the crucial role of dendritic dimensions in morphological classification, we further conducted morphometric analysis by measuring the extents of the dendritic trees of neurons from the two groups. The mean rostro-caudal dendritic lengths for neurons with or without RD were 513.78 ± 56.95 μm and 361.88 ± 31.34 μm, respectively, and these were significantly different (*p* = 0.029). A similar comparison indicated that dendritic extents in the dorsal-ventral axis did not differ significantly between the two subpopulations (190.81 ± 24.12 μm vs 156.85 ± 21.62 μm, *p* = 0.305) (Table 3).

**TABLE 3:** Morphological dimensions of dendrites measured from SG neurons with or without RD.

Parameter	Neurons without RD (*n* = 16)	Neurons with RD (*n* = 17)	*p* Value
RC (μm)	361.88 ± 31.34	513.78 ± 56.95*	0.029
DV (μm)	156.85 ± 21.62	190.81 ± 24.12	0.305
SR (μm)	165.63 ± 17.51	269.00 ± 19.37***	<0.001
SC(μm)	196.25 ± 19.71	244.78 ± 45.39	0.736
SD (μm)	68.78 ± 11.25	71.35 ± 14.69	0.599
SV (μm)	88.07 ± 13.15	119.46 ± 12.93	0.099

*Data reported as mean ± SEM. RC, rostro-caudal extent of dentrites; DV, dorsal-ventral expansion of dendrites; SR, SC, SD, SV, dendrites spread from center of the soma to rostral, caudal, dorsal, and ventral limit, respectively.*

*^∗^p < 0.05.*

*^∗∗∗^p < 0.001.*

## Discussion

The aim of the present descriptive study is to define the electrophysiological and morphological characteristics of SG neurons with RD, and several key conclusions can be drawn from this study. First, compared to RD-lacking SG neurons, RD-expressing neurons showed distinctive electrophysiological signatures including more depolarized RMP, smaller Rin, more hyperpolarized AP thresholds, and increased spike frequencies, representing a higher membrane excitability. Meanwhile, SG neurons with RD predominantly exhibit a tonic firing pattern. Second, we found that I_*T*_ was a vital ionic mechanism basis responsible for the generation of RD in SG, while the onset of RD was regulated by I_*h*_. Third, neurons with RD receive monosynaptic as well as polysynaptic excitatory inputs from both Aδ and C afferents, and they showed lower frequencies regarding sEPSCs together with mEPSCs than neurons without RD. Finally, we found anatomical diversity in RD-expressing neurons, with islet cells constituting half of the population.

### SG Neurons With RD Exhibit Distinct Intrinsic Neuronal Characteristics

Recent studies have linked I_*h*_/I_*T*_ to neuronal electrical properties. For instance, intracellular recording from primary sensory neurons found that I_*h*_ blockage by ZD7288 changed RMP toward the negative direction, prolonged AP duration, and resulted in diminished effects on AP frequency (Hogan and Poroli, 2008). In our prior investigations, we have noted that impeding I_*h*_ by using drugs interfering with the function of HCN channels robustly decreased firing rate, delayed RD latency, and lower post-hyperpolarization spike frequency in SG neurons, indicating an excitatory influence of I_*h*_ (Liu et al., 2015; Hu et al., 2016). Likewise, a number of groups have suggested the role of I_*T*_ in establishing intrinsic excitability. We have shown previously that there were remarkable differences in properties of SG neurons with or without I_*T*_. For I_*T*_-expressing neurons, they displayed a more hyperpolarized AP threshold, a smaller difference of AP threshold and RMP, as well as elevated firing frequency (Wu et al., 2018). Meanwhile, a recent study also showed that AP amplitude was decreased in Cav3.2-ablated SG neurons (Candelas et al., 2019). Thus, as suggested by identifying I_*h*_/I_*T*_ in RD-expressing SG neurons, the intrinsic excitability of this population might involve a contribution from these two subthreshold currents.

Previous work on deep dorsal horn neurons, which are also of paramount importance for integrating somatosensory information, found that AP threshold was more hyperpolarized and the difference of AP threshold and RMP tended to be smaller in neurons with a high-amplitude RD; while RMP, Rin and AP amplitude in RD-expressing deep dorsal horn neurons were comparable to those lacking it (Rivera-Arconada and Lopez-Garcia, 2015). Here, marked differences in active and passive membrane properties were identified in SG neurons with or without RD. In line with the aforementioned study from the mouse deep dorsal horn, we found neurons with RD in superficial spinal dorsal horn exhibited significantly lower AP threshold and smaller difference of AP threshold and RMP. However, we also observed a significant difference in RMP, Rin, AP duration along with AP amplitude between the two groups. The differences in our observations raise the possibility that the intrinsic excitability of different spinal cord neurons may correlate with RD in unique ways. Besides, we found tonic-firing neurons with RD generated considerably increased spike frequencies in response to defined depolarizing currents. Together, these data indicate that SG neurons with RD display higher membrane excitability.

Additional support for the relationship between RD and neuron excitability came from the result of the spike adaption index. Diverse degrees of firing adaptation have been observed in SG neurons (Ruscheweyh and Sandkuhler, 2002; Olschewski et al., 2009; Melnick, 2011). An interesting study analyzed the major factors determining the appearance of spike frequency adaption in SG neurons and found that lower Na^+^ conductance was critical for the generation of adapting firing (Melnick et al., 2004). Given that RD-expressing neurons exhibited stronger adaptation than RD-lacking neurons, the present study implies that the expression or function of Na^+^ channels may differ between these two populations. Moreover, due to neurons with strong adaptation have been suggested to be nociceptive neurons with specific cutaneous afferent input (Lopez-Garcia and King, 1994), it is reasonable to assume the importance of SG neurons with RD underpinning spinal sensory encoding.

### SG Neurons With RD Show Specific Cellular Distributions

The prevalence of rebound spikes in randomly sampled mouse SG neurons was around 30% according to previous reports (Tadros et al., 2012; Candelas et al., 2019). However, the percentage of RD in SG neurons raised to 44.44% in this study. Reasons for this incidence discrepancy may attribute to the different species we used in our studies. Furthermore, recent literature segregating SG neurons in a genetically-defined approach found two-thirds of cholinergic interneurons in mouse dorsal horn displayed RD following hyperpolarization (Mesnage et al., 2011), whilst the fraction of RD in delayed (short-latency) firing SG interneurons expressing neuropeptide Y (NPY) Y1 receptor from mice was 32% (Sinha et al., 2021). This quantitative difference suggests that RD might distribute in SG neurons in a specific cellular manner.

Substantia gelatinosa neurons are a very heterogeneous population of interneurons with varied electrophysiological features, morphologies, and molecular profiles (Lu et al., 2006; Todd, 2017; Peirs et al., 2020). SG neurons can be divided into five broad categories on structural grounds, namely islet, central, vertical, radial, and unclassified cells. A series of pioneering works well established that islet cells are predominantly inhibitory (Todd and Sullivan, 1990; Maxwell et al., 2007; Yasaka et al., 2010), while other morphologies have been proposed to be associated with both excitatory and inhibitory phenotypes in the literature (Hantman et al., 2004; Lu and Perl, 2005; Maxwell et al., 2007; Duan et al., 2014; Punnakkal et al., 2014; Smith et al., 2015; Boyle et al., 2019).

Given that the most common morphological type in our sample of SG neurons with RD was “islet,” and that the majority of these cells discharged tonically when depolarized, a vital characteristic of inhibitory neurons (Lu and Perl, 2003; Zheng et al., 2010), a major finding of this study is that RD-expressing interneurons in SG have probably an inhibitory nature. However, a strict conclusion could finally be drawn based on future immunocytochemical studies. Furthermore, combined with transgenic technologies, recent publications have added greatly to our understanding of the functional heterogeneity of inhibitory interneurons in the superficial spinal dorsal horn. So far, several molecularly distinct inhibitory populations in the superficial region of the dorsal spinal cord have been described according to neurochemical classification scheme established based on the expression of NPY, nitric oxide synthase (nNOS), parvalbumin (PV), galanin, calretinin (CR), and receptor tyrosine kinase (Tiong et al., 2011; Polgár et al., 2013; Smith et al., 2015; Cui et al., 2016; Iwagaki et al., 2016). Future work that identifies the expression of molecular compounds in RD-expressing SG neurons will be required to elucidate their exact neurochemical subtype and hence be useful for addressing their functional role in-depth.

### SG Neurons With RD Have Differential Excitatory Synaptic Inputs and Primary Afferent Innervations

Prior anatomical work on SG neurons from both rats and mice showed islet, central tonic, PV-positive, and CR-positive cells, which were identified with an inhibitory phenotype by morphology, electrophysiology, or the expression of particular molecular markers, received relatively weak excitatory inputs, as assessed by low-frequency sEPSCs (Grudt and Perl, 2002; Hughes et al., 2012; Smith et al., 2015; Graham, 2020). In keeping with these findings, our observation of excitatory synaptic inputs revealed that although the amplitudes of excitatory events (sEPSCs and mEPSCs) were comparable between the two clusters of SG neurons, neurons with RD presented lower frequencies of both sEPSCs and mEPSCs. Thus, our results mirror these recent reports indicating that inhibitory interneurons receive a lower excitatory drive. Because functional dendritic spines are recognized as sites of presynaptic inputs (Alvarez and Sabatini, 2007), it is tempting to speculate that a possible cause of the phenomenon reported here is RD-expressing SG neurons in our sample may exhibit less dendritic spine density in spite of larger dendritic areas. Another potential source of inconsistency regarding the EPSC frequency is that the presynaptic release probabilities for neurons with or without RD from local excitatory interneurons may differ. Nevertheless, these possible mechanisms need to be further confirmed by dendritic spine analysis along with a paired patch-clamp recording.

Numerous studies have investigated synaptic input onto inhibitory populations in dorsal horn superficial layers, and interpretations from these datasets suggest that discrete inhibitory SG interneurons have differing synaptic afferents. One of the previous studies from rats showed that islet cell normally received monosynaptic excitatory input from unmyelinated afferents (C afferents), this result was supported by an *in vitro* electrophysiological experiment using mice where NPY-expressing neurons displayed responses to monosynaptic inputs originated from C fibers (Grudt and Perl, 2002; Iwagaki et al., 2016). In contrast, immunolabelling work from Hughes et al. found that PV-immunoreactive cells, a subpopulation of islet neurons, were directly associated with VGLUT1-expressing terminals derived from myelinated afferent fibers, including both Aδ and Aβ fibers specifically (Hughes et al., 2012). Other inhibitory neurons belonging to the galanin or nNOS populations were likely to form contacts from both myelinated and unmyelinated classes of primary afferent either directly or indirectly, with many cells receiving convergent inputs (Hantman et al., 2004; Ganley et al., 2015). Although evidence from different groups showed disagreement about the primary innervations onto inhibitory SG neurons, the above evidence demonstrated that SG neurons with an inhibitory identity were likely recruited in gating innocuous as well as noxious information. In agreement with results reported by Ganley et al. and Hantman et al., we found that SG neurons with RD received both monosynaptic and polysynaptic inputs from Aδ and C fibers, the presumed nociceptive primary afferents. As we proposed that RD-expressing neurons were inhibitory interneurons based on their morphological and electrophysiological features, they may counterbalance excitatory drive through feedforward, feedback, or lateral inhibition in dorsal horn circuits, and hence contribute to nociceptive sensation and modulation.

### RD in SG Neurons Is Regulated by I_*h*_ and I_*T*_

Although several channels, for instance, inwardly-rectifying potassium channels (Wang et al., 2016), low-threshold TTX-resistant sodium channels (Kurowski et al., 2018), persistent sodium channels (Sangrey and Jaeger, 2010), and high-threshold calcium channels (Zheng and Raman, 2009) have been confirmed to be involved in regulating RD by hyperpolarizing RMP, decreasing neuronal input resistances, or enhancing synaptic inhibition that contributes to post-inhibitory depolarizations, currents mediated by HCN channels and T-type calcium channels are recognized as pivotal ionic factors underlying RD (Boehme et al., 2011; Engbers et al., 2011). With respect to the role of I_*T*_ in RD, pharmacological blockade of T-type calcium channels by using blockers such as NiCl_2_, mibefradil, or NNC 55-0396 could produce a significant block on the total number of spikes generated in RD (Alvina et al., 2009). In addition, in keeping with a recent study reporting that RD was barely detected in SG neurons without I_*T*_ (Wu et al., 2018), data from Candelas et al. also found that ablation of Cav3.2 but not Cav3.1 downregulated the proportion of RD in SG (Candelas et al., 2019). On the other hand, I_*h*_ has been proposed to be a key factor in determining the initiation of the first spike in RD, as both pharmacological blockade and genetic knockout of HCN2 could considerably delay RD latency (Zhu et al., 2018).

It has been demonstrated that various isoforms of T-type calcium channels along with HCN channels are present in SG (Peng et al., 2017; Candelas et al., 2019; Cheng et al., 2019). Alterations in the expression and function of these channels are responsible for the development and maintenance of chronic pain (Papp et al., 2010; Feng et al., 2019). Therefore, it is possible to assume a potential effect of RD on nociceptive processing. In the present work, we also found that abolishment of I_*T*_ significantly reduced the RD amplitude, while I_*h*_ blockade modulated the onset of the rebound firing. Our observations are in line with preceding reports showing the role of I_*h*_ and I_*T*_ in controlling rebound spike responses.

### Functional Implications of RD in SG Neurons

It has been postulated that as an intrinsic biophysical property in the sensory system, RD may play a possible role in encoding stimulus intensity (Large and Crawford, 2002; Oswald et al., 2004; Rivera-Arconada and Lopez-Garcia, 2015). As indicated by pilot observations, different firing patterns were linked with distinct neuronal functions. For instance, neurons showing tonic firing are proposed to be involved in encoding both the strength and duration of input signals. In addition to tonic-firing, initial-firing neurons also contribute to encoding the intensity of afferent excitation (Ruscheweyh and Sandkuhler, 2002). Thus, as the tonic and initial firing were the top two patterns recorded in RD-expressing SG neurons, our results indicate that this group of cells plays a substantial role in coding the intensity of ascending impulses. Additionally, RD has also been speculated to be an inhibition-excitation converter that transforms an inhibitory input into excitatory output (Sanchez-Vives and McCormick, 2000; Surges et al., 2006). However, our results only provide information about excitatory inputs onto RD-expressing neurons; future studies addressing their presynaptic inhibitory circuits involving primary afferents may further constitute circumstantial evidence in favor of this possibility.

## Conclusion

In summary, our study revealed that SG neurons with RD are not only electrophysiologically distinct from those lacking RD, but also differ in synaptic transmission and thus may play a unique physiological role in the nociception network.

## Data Availability Statement

The original contributions presented in the study are included in the article/Supplementary Material, further inquiries can be directed to the corresponding authors.

## Ethics Statement

The animal study was reviewed and approved by Institutional Animal Care and Use Committee of Nanchang University.

## Author Contributions

TL and DZ interpreted the data and designed and supervised the study. MZ and TL drafted and edited the manuscript. MZ and YY performed the electrophysiological and morphological experiments. XC, FZ, GX, WS, FL, and LL assisted in analyzing and interpreting the data. ZW, YZ, and XZ provided discussion in study design and interpretation of data. All authors read and approved the final manuscript.

## Conflict of Interest

The authors declare that the research was conducted in the absence of any commercial or financial relationships that could be construed as a potential conflict of interest.

## Publisher’s Note

All claims expressed in this article are solely those of the authors and do not necessarily represent those of their affiliated organizations, or those of the publisher, the editors and the reviewers. Any product that may be evaluated in this article, or claim that may be made by its manufacturer, is not guaranteed or endorsed by the publisher.

## References

[B1] Alvarez V. A. Sabatini B. L. (2007). Anatomical and physiological plasticity of dendritic spines. *Annu. Rev. Neurosci.* 30 79–97. 10.1146/annurev.neuro.30.051606.094222 17280523

[B2] Alvina K. Ellis-Davies G. Khodakhah K. (2009). T-type calcium channels mediate rebound firing in intact deep cerebellar neurons. *Neuroscience* 158 635–641. 10.1016/j.neuroscience.2008.09.052 18983899 PMC2649676

[B3] Balasubramanyan S. Stemkowski P. L. Stebbing M. J. Smith P. A. (2006). Sciatic chronic constriction injury produces cell-type-specific changes in the electrophysiological properties of rat substantia gelatinosa neurons. *J. Neurophysiol.* 96 579–590. 10.1152/jn.00087.2006 16611846

[B4] Boehme R. Uebele V. N. Renger J. J. Pedroarena C. (2011). Rebound excitation triggered by synaptic inhibition in cerebellar nuclear neurons is suppressed by selective T-type calcium channel block. *J. Neurophysiol.* 106 2653–2661. 10.1152/jn.00612.2011 21849607

[B5] Boyle K. A. Gradwell M. A. Yasaka T. Dickie A. C. Polgár E. Ganley R. P. (2019). Defining a spinal microcircuit that gates myelinated afferent input: implications for tactile allodynia. *Cell. Rep.* 28 526–540. 10.1016/j.celrep.2019.06.040 31291586 PMC6635381

[B6] Candelas M. Reynders A. Arango-Lievano M. Neumayer C. Fruquiere A. Demes E. (2019). Cav3.2 T-type calcium channels shape electrical firing in mouse Lamina II neurons. *Sci. Rep.* 9:3112. 10.1038/s41598-019-39703-3 30816223 PMC6395820

[B7] Cheng X. E. Ma L. X. Feng X. J. Zhu M. Y. Zhang D. Y. Xu L. L. (2019). Antigen retrieval pre-treatment causes a different expression pattern of Cav3.2 in rat and mouse spinal dorsal horn. *Eur. J. Histochem.* 63:2988. 10.4081/ejh.2019.2988 30678436 PMC6346256

[B8] Cui L. Miao X. Liang L. Abdus-Saboor I. Olson W. Fleming M. S. (2016). Identification of early RET+ deep dorsal spinal cord interneurons in gating pain. *Neuron* 91:1413. 10.1016/j.neuron.2016.09.010 27657453

[B9] Duan B. Cheng L. Bourane S. Britz O. Padilla C. Garcia-Campmany L. (2014). Identification of spinal circuits transmitting and gating mechanical pain. *Cell* 159 1417–1432. 10.1016/j.cell.2014.11.003 25467445 PMC4258511

[B10] Duan B. Cheng L. Ma Q. (2018). Spinal circuits transmitting mechanical pain and itch. *Neurosci. Bull.* 34 186–193. 10.1007/s12264-017-0136-z 28484964 PMC5799122

[B11] Engbers J. D. Anderson D. Tadayonnejad R. Mehaffey W. H. Molineux M. L. Turner R. W. (2011). Distinct roles for I(T) and I(H) in controlling the frequency and timing of rebound spike responses. *J. Physiol.* 589 5391–5413. 10.1113/jphysiol.2011.215632 21969455 PMC3240880

[B12] Feng X. J. Ma L. X. Jiao C. Kuang H. X. Zeng F. Zhou X. Y. (2019). Nerve injury elevates functional Cav3.2 channels in superficial spinal dorsal horn. *Mol. Pain* 15:1744806919836569. 10.1177/1744806919836569 30803310 PMC6458665

[B13] Ganley R. P. Iwagaki N. del Rio P. Baseer N. Dickie A. C. Boyle K. A. (2015). Inhibitory interneurons that express GFP in the PrP-GFP mouse spinal cord are morphologically heterogeneous, innervated by several classes of primary afferent and include Lamina I projection neurons among their postsynaptic targets. *J. Neurosci.* 35 7626–7642. 10.1523/jneurosci.0406-15.2015 25972186 PMC4429159

[B14] Graham B. A. (2020). Transgenic cross-referencing of inhibitory and excitatory interneuron populations to dissect neuronal heterogeneity in the dorsal horn. *Front. Mol. Neurosci.* 13:32. 10.3389/fnmol.2020.00032 32362812 PMC7180513

[B15] Graham B. A. Hughes D. I. (2020). Defining populations of dorsal horn interneurons. *Pain* 161 2434–2436. 10.1097/j.pain.0000000000002067 33065697 PMC7566298

[B16] Grudt T. J. Perl E. R. (2002). Correlations between neuronal morphology and electrophysiological features in the rodent superficial dorsal horn. *J. Physiol.* 540 189–207. 10.1113/jphysiol.2001.012890 11927679 PMC2290200

[B17] Ha G. E. Lee J. Kwak H. Song K. Kwon J. Jung S. Y. (2016). The Ca(2+)-activated chloride channel anoctamin-2 mediates spike-frequency adaptation and regulates sensory transmission in thalamocortical neurons. *Nat. Commun.* 7:13791. 10.1038/ncomms13791 27991499 PMC5187435

[B18] Hantman A. W. van den Pol A. N. Perl E. R. (2004). Morphological and physiological features of a set of spinal substantia gelatinosa neurons defined by green fluorescent protein expression. *J. Neurosci.* 24 836–842. 10.1523/JNEUROSCI.4221-03.2004 14749428 PMC6729829

[B19] Hogan Q. H. Poroli M. (2008). Hyperpolarization-activated current (I(h)) contributes to excitability of primary sensory neurons in rats. *Brain Res.* 1207 102–110. 10.1016/j.brainres.2008.02.066 18377879 PMC2745653

[B20] Hu T. Liu N. N. Lv M. H. Ma L. X. Peng H. Z. Peng S. C. (2016). Lidocaine inhibits HCN currents in rat spinal substantia gelatinosa neurons. *Anesth. Analg.* 122 1048–1059. 10.1213/ANE.0000000000001140 26756913 PMC4791316

[B21] Hughes D. I. Sikander S. Kinnon C. M. Boyle K. A. Watanabe M. Callister R. J. (2012). Morphological, neurochemical and electrophysiological features of parvalbumin-expressing cells: a likely source of axo-axonic inputs in the mouse spinal dorsal horn. *J. Physiol.* 590 3927–3951. 10.1113/jphysiol.2012.235655 22674718 PMC3476641

[B22] Iwagaki N. Ganley R. P. Dickie A. C. Polgár E. Hughes D. I. Del Rio P. (2016). A combined electrophysiological and morphological study of neuropeptide Y-expressing inhibitory interneurons in the spinal dorsal horn of the mouse. *Pain* 157 598–612. 10.1097/j.pain.0000000000000407 26882346 PMC4751741

[B23] Kuner R. (2010). Central mechanisms of pathological pain. *Nat. Med.* 16 1258–1266. 10.1038/nm.2231 20948531

[B24] Kurowski P. Grzelka K. Szulczyk P. (2018). Ionic mechanism underlying rebound depolarization in medial prefrontal cortex pyramidal neurons. *Front. Cell. Neurosci.* 12:93. 10.3389/fncel.2018.00093 29740284 PMC5924806

[B25] Large E. W. Crawford J. D. (2002). Auditory temporal computation: interval selectivity based on post-inhibitory rebound. *J. Comput. Neurosci.* 13 125–142. 10.1023/a:1020162207511 12215726

[B26] Liu N. N. Zhang D. Y. Zhu M. Y. Luo S. W. Liu T. (2015). Minocycline inhibits hyperpolarization-activated currents in rat substantia gelatinosa neurons. *Neuropharmacology* 95 110–120. 10.1016/j.neuropharm.2015.03.001 25777286

[B27] Lopez-Garcia J. A. King A. E. (1994). Membrane properties of physiologically classified rat dorsal horn neurons in vitro: correlation with cutaneous sensory afferent input. *Eur. J. Neurosci.* 6 998–1007. 10.1111/j.1460-9568.1994.tb00594.x 7952286

[B28] Lu V. B. Moran T. D. Balasubramanyan S. Alier K. A. Dryden W. F. Colmers W. F. (2006). Substantia Gelatinosa neurons in defined-medium organotypic slice culture are similar to those in acute slices from young adult rats. *Pain* 121 261–275. 10.1016/j.pain.2006.01.009 16516387

[B29] Lu Y. Perl E. R. (2003). A specific inhibitory pathway between substantia gelatinosa neurons receiving direct C-fiber input. *J. Neurosci.* 23 8752–8758. 10.1523/jneurosci.23-25-08752.2003 14507975 PMC6740424

[B30] Lu Y. Perl E. R. (2005). Modular organization of excitatory circuits between neurons of the spinal superficial dorsal horn (laminae I and II). *J. Neurosci.* 25 3900–3907. 10.1523/jneurosci.0102-05.2005 15829642 PMC6724918

[B31] Maxwell D. J. Belle M. D. Cheunsuang O. Stewart A. Morris R. (2007). Morphology of inhibitory and excitatory interneurons in superficial laminae of the rat dorsal horn. *J. Physiol.* 584 521–533. 10.1113/jphysiol.2007.140996 17717012 PMC2277171

[B32] Melnick I. V. (2011). A-type K+ current dominates somatic excitability of delayed firing neurons in rat substantia gelatinosa. *Synapse* 65 601–607. 10.1002/syn.20879 21484879

[B33] Melnick I. V. Santos S. F. Safronov B. V. (2004). Mechanism of spike frequency adaptation in substantia gelatinosa neurones of rat. *J. Physiol.* 559 383–395. 10.1113/jphysiol.2004.066415 15235088 PMC1665127

[B34] Mesnage B. Gaillard S. Godin A. G. Rodeau J. L. Hammer M. Von Engelhardt J. (2011). Morphological and functional characterization of cholinergic interneurons in the dorsal horn of the mouse spinal cord. *J. Comp. Neurol.* 519 3139–3158. 10.1002/cne.22668 21618225

[B35] Olschewski A. Schnoebel-Ehehalt R. Li Y. Tang B. Bräu M. E. Wolff M. (2009). Mexiletine and lidocaine suppress the excitability of dorsal horn neurons. *Anesth. Analg.* 109 258–264. 10.1213/ane.0b013e3181a3d5d8 19535719

[B36] Oswald A. M. Chacron M. J. Doiron B. Bastian J. Maler L. (2004). Parallel processing of sensory input by bursts and isolated spikes. *J. Neurosci.* 24 4351–4362. 10.1523/JNEUROSCI.0459-04.2004 15128849 PMC6729439

[B37] Papp I. Holló K. Antal M. (2010). Plasticity of hyperpolarization-activated and cyclic nucleotid-gated cation channel subunit 2 expression in the spinal dorsal horn in inflammatory pain. *Eur. J. Neurosci.* 32 1193–1201. 10.1111/j.1460-9568.2010.07370.x 20726890

[B38] Pedroarena C. M. (2010). Mechanisms supporting transfer of inhibitory signals into the spike output of spontaneously firing cerebellar nuclear neurons in vitro. *Cerebellum* 9 67–76. 10.1007/s12311-009-0153-1 20148319

[B39] Peirs C. Dallel R. Todd A. J. (2020). Recent advances in our understanding of the organization of dorsal horn neuron populations and their contribution to cutaneous mechanical allodynia. *J. Neural. Transm. (Vienna)* 127 505–525. 10.1007/s00702-020-02159-1 32239353 PMC7148279

[B40] Peng S. C. Wu J. Zhang D. Y. Jiang C. Y. Xie C. N. Liu T. (2017). Contribution of presynaptic HCN channels to excitatory inputs of spinal substantia gelatinosa neurons. *Neuroscience* 358 146–157. 10.1016/j.neuroscience.2017.06.046 28673721

[B41] Polgár E. Sardella T. C. P. Tiong S. Y. X. Locke S. Watanabe M. Todd A. J. (2013). Functional differences between neurochemically defined populations of inhibitory interneurons in the rat spinal dorsal horn. *Pain* 154 2606–2615. 10.1016/j.pain.2013.05.001 23707280 PMC3858808

[B42] Punnakkal P. von Schoultz C. Haenraets K. Wildner H. Zeilhofer H. U. (2014). Morphological, biophysical and synaptic properties of glutamatergic neurons of the mouse spinal dorsal horn. *J. Physiol.* 592 759–776. 10.1113/jphysiol.2013.264937 24324003 PMC3934713

[B43] Rivera-Arconada I. Lopez-Garcia J. A. (2015). Characterisation of rebound depolarisation in mice deep dorsal horn neurons in vitro. *Pflugers Arch.* 467 1985–1996. 10.1007/s00424-014-1623-y 25292284

[B44] Ruscheweyh R. Sandkuhler J. (2002). Lamina-specific membrane and discharge properties of rat spinal dorsal horn neurones in vitro. *J. Physiol.* 541 231–244. 10.1113/jphysiol.2002.017756 12015432 PMC2290304

[B45] Sanchez-Vives M. V. McCormick D. A. (2000). Cellular and network mechanisms of rhythmic recurrent activity in neocortex. *Nat. Neurosci.* 3 1027–1034. 10.1038/79848 11017176

[B46] Sangrey T. Jaeger D. (2010). Analysis of distinct short and prolonged components in rebound spiking of deep cerebellar nucleus neurons. *Eur. J. Neurosci.* 32 1646–1657. 10.1111/j.1460-9568.2010.07408.x 21039958 PMC3058674

[B47] Sinha G. P. Prasoon P. Smith B. N. Taylor B. K. (2021). Fast A-type currents shape a rapidly adapting form of delayed short latency firing of excitatory superficial dorsal horn neurons that express the NPY Y1 receptor. *J. Physiol.* 599 2723–2750. 10.1113/jp281033 33768539 PMC9583652

[B48] Smith K. M. Boyle K. A. Madden J. F. Dickinson S. A. Jobling P. Callister R. J. (2015). Functional heterogeneity of calretinin-expressing neurons in the mouse superficial dorsal horn: implications for spinal pain processing. *J. Physiol.* 593 4319–4339. 10.1113/JP270855 26136181 PMC4594251

[B49] Sun H. Zhang H. Ross A. Wang T. T. Al-Chami A. Wu S. H. (2020). Developmentally regulated rebound depolarization enhances spike timing precision in auditory midbrain neurons. *Front. Cell. Neurosci.* 14:236. 10.3389/fncel.2020.00236 32848625 PMC7424072

[B50] Surges R. Sarvari M. Steffens M. Els T. (2006). Characterization of rebound depolarization in hippocampal neurons. *Biochem. Biophys. Res. Commun.* 348 1343–1349. 10.1016/j.bbrc.2006.07.193 16925982

[B51] Tadayonnejad R. Mehaffey W. H. Anderson D. Turner R. W. (2009). Reliability of triggering postinhibitory rebound bursts in deep cerebellar neurons. *Channels (Austin)* 3 149–155. 10.4161/chan.3.3.8872 19535914

[B52] Tadros M. A. Harris B. M. Anderson W. B. Brichta A. M. Graham B. A. Callister R. J. (2012). Are all spinal segments equal: intrinsic membrane properties of superficial dorsal horn neurons in the developing and mature mouse spinal cord. *J. Physiol.* 590 2409–2425. 10.1113/jphysiol.2012.227389 22351631 PMC3424761

[B53] Tiong S. Y. Polgár E. van Kralingen J. C. Watanabe M. Todd A. J. (2011). Galanin-immunoreactivity identifies a distinct population of inhibitory interneurons in laminae I-III of the rat spinal cord. *Mol. Pain* 7:36. 10.1186/1744-8069-7-36 21569622 PMC3118366

[B54] Todd A. J. (2010). Neuronal circuitry for pain processing in the dorsal horn. *Nat. Rev. Neurosci.* 11 823–836. 10.1038/nrn2947 21068766 PMC3277941

[B55] Todd A. J. (2017). Identifying functional populations among the interneurons in laminae I-III of the spinal dorsal horn. *Mol. Pain* 13:1744806917693003. 10.1177/1744806917693003 28326935 PMC5315367

[B56] Todd A. J. Sullivan A. C. (1990). Light microscope study of the coexistence of GABA-like and glycine-like immunoreactivities in the spinal cord of the rat. *J. Comp. Neurol.* 296 496–505. 10.1002/cne.902960312 2358549

[B57] Wang X. X. Jin Y. Sun H. Ma C. Zhang J. Wang M. (2016). Characterization of rebound depolarization in neurons of the rat medial geniculate body in vitro. *Neurosci. Bull.* 32 16–26. 10.1007/s12264-015-0006-5 26781877 PMC5563751

[B58] Wu J. Peng S. C. Xiao L. H. Cheng X. E. Kuang H. X. Zhu M. Y. (2018). Cell-type specific distribution of T-Type calcium currents in Lamina II neurons of the rat spinal cord. *Front. Cell. Neurosci.* 12:370. 10.3389/fncel.2018.00370 30386213 PMC6199353

[B59] Yasaka T. Kato G. Furue H. Rashid M. H. Sonohata M. Tamae A. (2007). Cell-type-specific excitatory and inhibitory circuits involving primary afferents in the rat spinal dorsal horn in vitro. *J. Physiol.* 581 603–618. 10.1113/jphysiol.2006.123919 17347278 PMC2075204

[B60] Yasaka T. Tiong S. Y. Hughes D. I. Riddell J. S. Todd A. J. (2010). Populations of inhibitory and excitatory interneurons in lamina II of the adult rat spinal dorsal horn revealed by a combined electrophysiological and anatomical approach. *Pain* 151 475–488. 10.1016/j.pain.2010.08.008 20817353 PMC3170912

[B61] Zhang C. Tonsfeldt K. J. Qiu J. Bosch M. A. Kobayashi K. Steiner R. A. (2013). Molecular mechanisms that drive estradiol-dependent burst firing of Kiss1 neurons in the rostral periventricular preoptic area. *Am. J. Physiol. Endocrinol. Metab.* 305 E1384–E1397. 10.1152/ajpendo.00406.2013 24105416 PMC3882370

[B62] Zheng J. Lu Y. Perl E. R. (2010). Inhibitory neurones of the spinal substantia gelatinosa mediate interaction of signals from primary afferents. *J. Physiol.* 588 2065–2075. 10.1113/jphysiol.2010.188052 20403977 PMC2911212

[B63] Zheng N. Raman I. M. (2009). Ca currents activated by spontaneous firing and synaptic disinhibition in neurons of the cerebellar nuclei. *J. Neurosci.* 29 9826–9838. 10.1523/JNEUROSCI.2069-09.2009 19657035 PMC2746933

[B64] Zhu M. Y. Idikuda V. K. Wang J. B. Wei F. S. Kumar V. Shah N. (2018). Shank3-deficient thalamocortical neurons show HCN channelopathy and alterations in intrinsic electrical properties. *J. Physiol.* 596 1259–1276. 10.1113/jp275147 29327340 PMC5878232

[B65] Zhu M. Y. Zhang D. Y. Peng S. C. Liu N. N. Wu J. Kuang H. X. (2019). Preparation of acute spinal cord slices for whole-cell patch-clamp recording in substantia gelatinosa neurons. *J. Vis. Exp.* 143:e58479. 10.3791/58479 30735185

