# Supplementary material for: Electrophysiological and Morphological Features of Rebound Depolarization Characterized Interneurons in Rat Superficial Spinal Dorsal Horn
Source: Front Cell Neurosci. 2021 Sep 21;15:736879. doi: 10.3389/fncel.2021.736879 (PMC8490703; doi:10.3389/fncel.2021.736879)
Supplement: Supplementary file 1 [file Data_Sheet_1.DOCX]

Supplementary Material

Electrophysiological and morphological features of rebound depolarization characterized interneurons in rat superficial spinal dorsal horn

**Mengye Zhu^1,2†^, Yi Yan^1,2†^, Xuezhong Cao^1,2^, Fei Zeng^1,2^, Gang Xu^1,2^, Wei Shen^1,2^, Fan Li^1,2^, Lingyun Luo^1,2^, Zhijian Wang^1,2^, Yong Zhang^1,2^, Xuexue Zhang^1,2^, Daying Zhang^1,2*^ AND Tao Liu^3*^**

^1^ Department of Pain Medicine, the First Affiliated Hospital of Nanchang University, Nanchang, Jiangxi 330006, China,

^2^ Institute of Pain Medicine, Jiangxi Academy of Clinical and Medical Sciences, Nanchang, Jiangxi 330006, China,

^3^ Center for Experimental Medicine, the First Affiliated Hospital of Nanchang University, Nanchang, Jiangxi 330006, China

*** Correspondence:**Daying Zhang
zdysino@163.com

Tao Liu
liutao1241@ncu.edu.cn

## †These authors contributed equally to this work.


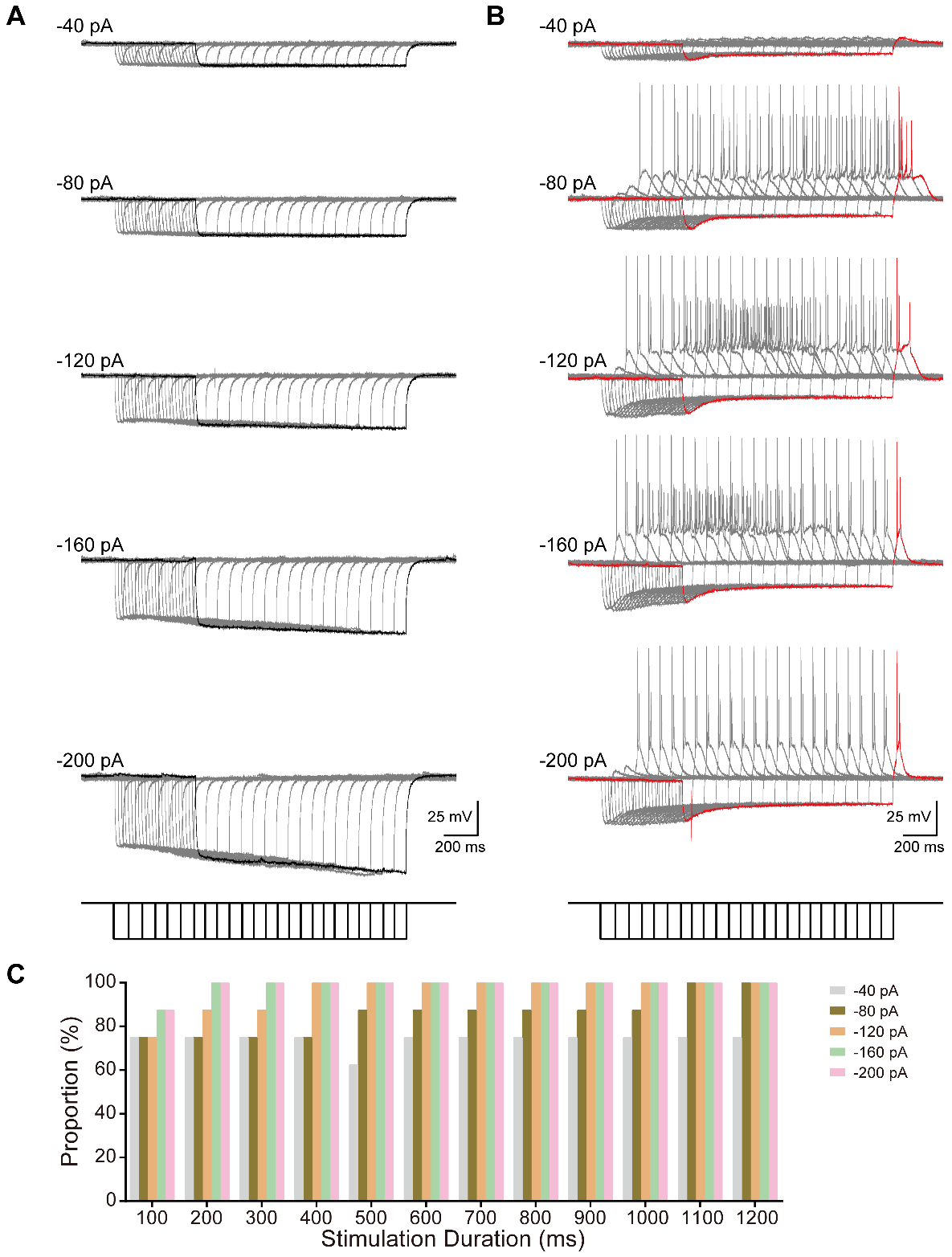


**Figure S1.** Responses of SG neurons with or without RD to hyperpolarizing currents of various magnitudes and durations.

(A) Representative traces of responses recorded from non-RD SG neurons to hyperpolarizing currents with durations ranged from 50 ms to 1200 ms (50 ms/step). Neither raising injected hyperpolarizing current nor increasing the duration of hyperpolarizing current could trigger RD in this population.

(B) Sample traces of the RD behavior following hyperpolarizing currents of various magnitudes (-40 pA to -200 pA) and durations (50 ms to 1200 ms) in RD-expressing SG neurons.

(C) Summary bar graph showing proportions of RD-expressing neurons exhibiting RD (n = 8) in response to a series of hyperpolarizing currents. Quantitative results showed that a 400-ms hyperpolarizing current at -120 pA was sufficient to drive RD responses in all RD-expressing neurons.


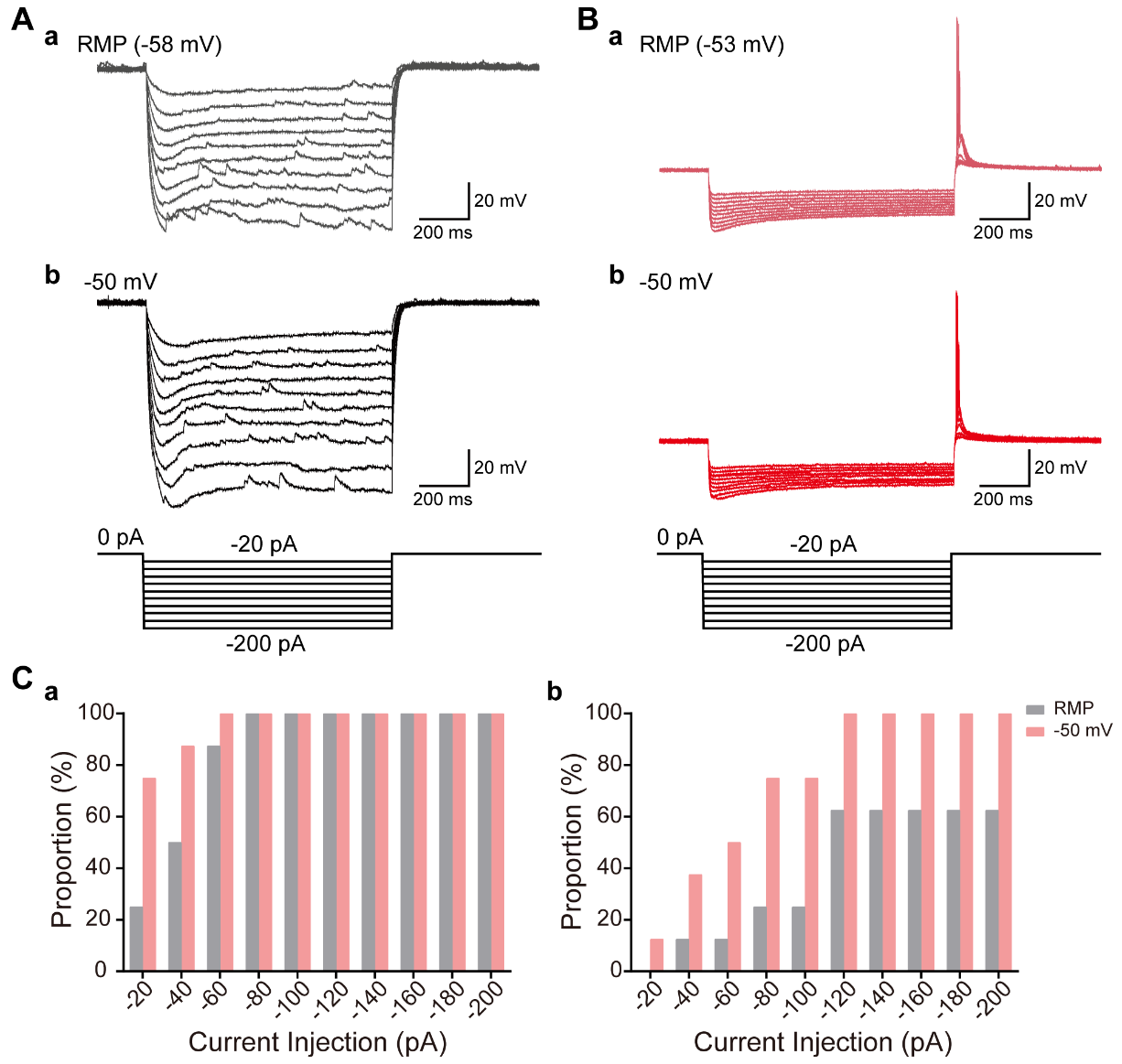


**Figure S2.** Responses of SG neurons with or without RD to hyperpolarizing currents at different holding potentials.

(A-B) Representative voltage responses to hyperpolarization currents steps (-20 - -200 pA in 20 pA decrements of 1000-ms duration) with holding potential set at -50 mV and RMP from neurons without (A) and with RD (B). Although depolarizing the holding potential from RMP to -50 mV facilitated RD discharge in RD-expressing neurons, it didn’t alter the responses of RD-lacking neurons to hyperpolarizing current stimulation.

(C) Summary bar graph showing proportions of neurons exhibiting RD (n = 8) in response to hyperpolarizing currents at different holding potential (gray: RMP, pink: -50 mV). Depolarized holding potential facilitated the generation of RD behaviors with hyperpolarizing currents at -20 pA to -60 pA, while hyperpolarizing currents at -80 pA to -200 pA could fully evoke RD regardless the holding potential.

(D) Histogram figure showing that depolarized holding potential facilitated the generation of RD discharge in SG neurons with RD (n = 8).


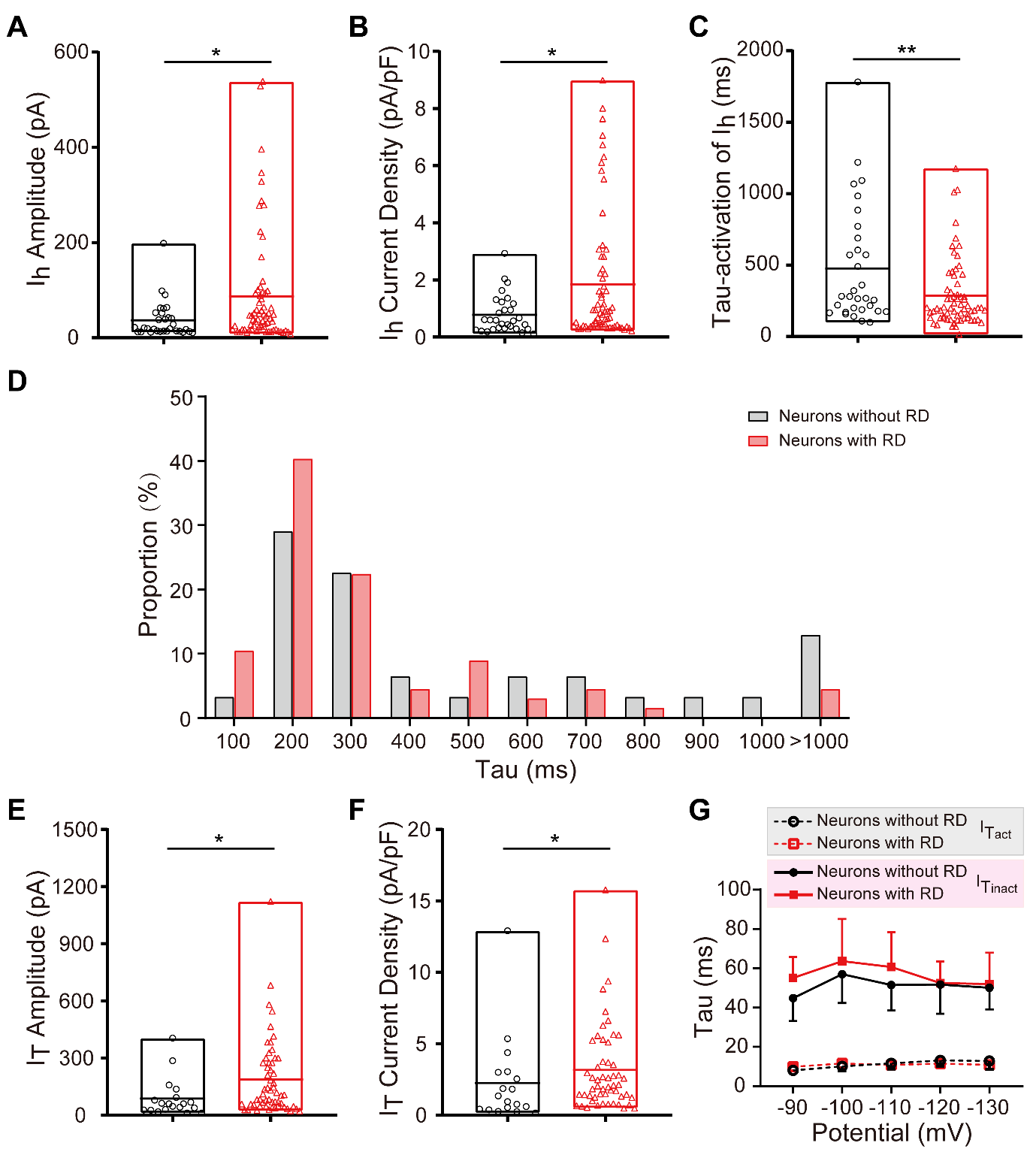


**Figure S3.** Amplitude and time constant of I_h_ and I_T_ in SG neurons with or without RD.

(A-C) Mean amplitude (A), averaged current density (B) and activation time constant (C) of I_h_ at -130 mV from SG neurons with (n = 66) or without RD (n = 27).

(D) Histogram figure showing tau-activation of I_h_ (at -130 mV) in the subtypes of SG neurons.

(E-G) Current amplitude (A) and density (B), activation and inactivation time constant (C) of I_T_ at -130 mV in RD (n = 51) and non-RD (n = 18) SG neurons.

**Table S1.** Averaged amplitude and time constant of I_h_ in SG neurons with or without RD with different discharge patterns

| **Group** | **Discharge pattern** | **Amplitude**  **(pA)** | **Time constant**  **(ms)** |
| --- | --- | --- | --- |
| Neurons without RD | Tonic (n = 11) | 29.64 ± 8.50 | 447.45 ± 100.18 |
|  | Single (n = 3) | 96.40 ± 51.23 | 454.86 ± 269.63 |
|  | Phasic (n = 4) | 24.02 ± 6.45 | 761.69 ± 301.40 |
|  | Initial (n = 7) | 39.72 ± 11.13 | 280.98 ± 72.75 |
| Neurons with RD | Tonic (n = 40) | 98.20 ± 21.99 | 256.00 ± 39.92 |
|  | Single (n = 3) | 91.87 ± 21.65 | 461.49 ± 111.42 |
|  | Phasic (n = 6) | 60.38 ± 23.83 | 315.85 ± 72.63 |
|  | Initial (n = 14) | 86.67 ± 28.16 | 324.96 ± 67.25 |

Values given as mean ± SEM. RD: rebound depolarization.

**Table S2.** Mean amplitude and time constant of I_T_ in SG neurons with or without RD with different discharge patterns

| **Group** | **Discharge pattern** | **Amplitude**  **(pA)** | **Tau I_Tact_**  **(ms)** | **Tau I_Tinact_**  **(ms)** |
| --- | --- | --- | --- | --- |
| Neurons without RD | Tonic (n = 12) | 237.33 ± 41.15 | 13.66 ± 2.36 | 65.69 ± 33.29 |
|  | Phasic (n = 3) | 100.11 ± 27.21 | 12.22 ± 4.14 | 40.19 ± 2.04 |
|  | Initial (n = 3) | 90.76 ± 25.62 | 6.35 ± 1.87 | 32.25 ± 6.09 |
| Neurons with RD | Tonic (n = 33) | 82.40 ± 31.42 | 11.75 ± 3.69 | 54.18 ± 16.22 |
|  | Phasic (n = 5) | 34.22 ± 12.73 | 6.94 ± 3.32 | 24.01 ± 10.59 |
|  | Initial (n = 9) | 91.60 ± 26.16 | 6.77 ± 4.08 | 66.52 ± 26.29 |

Values given as mean ± SEM. RD: rebound depolarization.

**Table S3.** Parameters of C-fiber mediated EPSCs in SG neurons with or without RD

| **Parameter** | **Group** | **Total** | **Number of Neurons** | ***P* value** |
| --- | --- | --- | --- | --- |
| Velocity (m/s) | Neurons without RD | 0.45 ± 0.06 | 20 | 0.165 |
|  | Neurons with RD | 0.58 ± 0.07 | 21 |  |
| Amplitude (pA) | Neurons without RD | 109.58 ± 14.62 | 19 | 0.698 |
|  | Neurons with RD | 121.52 ± 29.52 | 14 |  |
| Rise time (ms) | Neurons without RD | 8.03 ± 1.63 | 15 | 0.198 |
|  | Neurons with RD | 5.06 ± 1.22 | 10 |  |
| Decay time (ms) | Neurons without RD | 13.96 ± 1.77 | 18 | 0.533 |
|  | Neurons with RD | 12.12 ± 2.42 | 13 |  |

Values given as mean ± SEM. RD: rebound depolarization.

**Table S4.** Parameters of Aδ-fiber evoked EPSCs in SG neurons with or without RD

| **Parameter** | **Group** | **Total** | **Number of Neurons** | ***P* value** |
| --- | --- | --- | --- | --- |
| Velocity (m/s) | Neurons without RD | 2.12 ± 0.35 | 15 | 0.829 |
|  | Neurons with RD | 2.21 ± 0.21 | 15 |  |
| Amplitude (pA) | Neurons without RD | 86.86 ± 17.95 | 14 | 0.003 |
|  | Neurons with RD | 235.04 ± 43.15^**^ | 12 |  |
| Rise time (ms) | Neurons without RD | 5.48 ± 1.18 | 13 | 0.108 |
|  | Neurons with RD | 3.26 ± 0.53 | 12 |  |
| Decay time (ms) | Neurons without RD | 21.23 ± 8.76 | 10 | 0.741 |
|  | Neurons with RD | 17.59 ± 5.92 | 9 |  |

Values given as mean ± SEM. RD: rebound depolarization. ***p* < 0.01.
